# Numeric aspects in pitch identification: an fMRI study

**DOI:** 10.1186/1471-2202-12-26

**Published:** 2011-03-09

**Authors:** Michael Schwenzer, Klaus Mathiak

**Affiliations:** 1 Department of Psychiatry, Psychotherapy and Psychosomatics, RWTH Aachen University, Aachen, Germany; 2 Interdisciplinary Centre for Clinical Research (IZKF), RWTH Aachen, University, Aachen, Germany; 3 JARA - Translational Brain Medicine, Aachen, Germany; 4 Institute of Neuroscience and Medicine (INM-1), Research Centre Jülich, Jülich, Germany

## Abstract

**Background:**

Pitch identification had yielded unique response patterns compared to other auditory skills. Selecting one out of numerous pitches distinguished this task from detecting a pitch ascent. Encoding of numerous stimuli had activated the intraparietal sulcus in the visual domain. Therefore, we hypothesized that numerosity encoding during pitch identification activates the intraparietal sulcus as well.

**Methods:**

To assess pitch identification, the participants had to recognize a single pitch from a set of four possible pitches in each trial. Functional magnetic resonance imaging (fMRI) disentangled neural activation during this four-pitch-choice task from activation during pitch contour perception, tone localization, and pitch discrimination.

**Results:**

Pitch identification induced bilateral activation in the intraparietal sulcus compared to pitch discrimination. Correct responses in pitch identification correlated with activation in the left intraparietal sulcus. Pitch contour perception activated the superior temporal gyrus conceivably due to the larger range of presented tones. The differentiation between pitch identification and tone localization failed. Activation in an ACC-hippocampus network distinguished pitch discrimination from pitch identification.

**Conclusion:**

Pitch identification is distinguishable from pitch discrimination on the base of activation in the IPS. IPS activity during pitch identification may be the auditory counterpart of numerosity encoding in the visual domain.

## Background

Pitch identification had yielded unique behavioral response patterns in comparison to other pitch perception skills [1-3]. The present study aimed to characterize the neurophysiologic process underlying pitch identification. Task-specific brain activation may validate the distinction of pitch identification from other auditory skills.

Identification requires the recognition of a stimulus from a set of many stimuli [4]. Previous research suggested that cognitive processing of many alternatives relies on the mental representation of numerosity [5-7]. Numerosity refers to the cardinal property of a set [5], e.g. *four *tones. In addition, representation on a continuous scale of pitches may support pitch identification in terms of magnitude processing [6-8] comparable to mental space [9,10].

In the visual domain, numerosity processing activated the intraparietal sulcus (IPS) [5,11-13]. In the auditory domain, however, research hardly considered a possible association of numerosity processing with the IPS. A report from Cusack (2005) [14] merits attention: when participants perceived interleaved tones as two segregated streams, activation in the IPS increased in comparison to the perception of one unified stream. This finding suggests that the recognition of a higher number of separate auditory stimuli called the same cerebral region than visual numerosity processing did. A higher number of stimuli and the representation of digits did not activate the IPS differentially suggesting that activation in the IPS reflects rather abstract encoding than variations in memory load [15].

Processing magnitude activated the intraparietal sulcus as well [5,11-13]. However, higher activation in the left than in the right IPS might differentiate numerosity from magnitude encoding because processing numbers but not length activated the left IPS [12,16]. This finding poses the question whether numerosity processing during pitch identification induces a cerebral lateralization effect.

Applying the same tasks as in previous behavioral and drug studies [2,17] should allow a preliminary evaluation whether behavioral performance, neurotransmission, and neuroanatomy correlate with each other. A four-choice reaction task assessed pitch identification. The participants performed three further tasks - pitch contour perception, tone localization, and pitch discrimination - to distinguish pitch identification from other auditory skills. In these tasks, the participants had to consider only a pitch ascent providing a lower number of mental alternatives than during pitch identification. In previous studies, performing the three control tasks was not specifically associated with IPS activity: Pitch contour perception activated the STG compared to a baseline condition [18-20]. Tone localization activated the posterior temporal lobe and parietal regions excluding the IPS [21-23]. Pitch discrimination relied on functions of the tonotopically organized Heschl's gyrus and of the planum temporale [24-26]. Nevertheless, there are little data on differential activation during pitch identification compared to other auditory skills.

This survey prompted the following hypothesis: pitch identification activates selectively the IPS - that has been associated with numerosity processing - as compared to pitch contour perception, tone localization, and pitch discrimination. Additionally we explored a possible lateralization effect to the left IPS during pitch identification.

## Methods

### Participants

Sixteen volunteers (eight males, eight females; age range 18-34 yrs.) participated. No participant was a professional musician according to an interview about musical expertise. No participant presented a medical or neurological dysfunction at clinical examination. Perception of tones with 440, 2000, or 4096 Hz frequencies below 20 dB SPL in the left or right ear in random order ensured normal hearing. All participants stated correctly on which side a tone occurred. All participants were right-handed as determined by scores above 65 in the Edinburgh Handedness Inventory [27]. All participants gave written informed consent prior to the examination. The ethics committee of the Medical Faculty of the University Tübingen approved the experiment. Research was carried out in compliance with the Helsinki Declaration.

### Stimuli and tasks

A standardization of the tests aimed to control some stimulus features that could increase brain activation: participants had to attend to pitch variations in all tasks. In each task, the criterion for the selection of the pitches had been to reach a medium up to high task difficulty to induce comparable attention and effort [2]. To implement a similar task difficulty, the pitch variations had to differ between the tasks - both similar difficulty and the same pitches in each task was not feasible. In order to achieve a similar difficulty despite different tonal arrangements, to support the participant to adhere differentially to each task, and to reduce habituation to a fixed pitch range, each task presented an individual set of pitches. Apart from the pitch identification task, the participants had to respond to a frequency ascent. The number of actually presented alternatives was high in each task: the participants had to respond to at least four kinds of trials. The different kinds of trials occurred in a pseudo-randomized order with the constriction that never the same kind of trial succeeded twice. The duration of overall tonal stimulation per trial was 200 ms with the exception of prolonged stimulation of 400 ms in the pitch contour perception task. The response keypad was four keys side by side, operated by index, middle, ring, and small finger of the right hand. The split-half reliability above .8 was good in previous versions of the tasks [2]. A high profile reliability above .6 (unpublished result) according the formula of Mosier [28] in a pilot study suggested that the tests assessed different skills.

During the pitch identification task, one of four frequencies (800, 832, 852, and 872 Hz) were played in each trial. The spacing between frequencies did not represent a standardized scale (e.g. musical, linear) to avoid a bias due to the recognition of regularity. In contrast to a 100 ms stimulation in previous experiments [2,17], tones lasted 200 ms to match the duration of stimulation to the other tasks. The participants memorized each frequency before measurement. The instruction told the participants to identify the tones independent from preceding trials. In addition, the scanner noise between the trials made it difficult to refer to a previous tone. The keys indicated rising frequencies from left through right, i.e. pressing the index finger after 800 Hz, pressing the middle finger after 832 Hz etc.

During pitch contour perception, the participants should detect whether or not a pitch ascended within a melody of descending pitches. To reduce the effect of musical pre-experience, the melodies resembled no established song. Each melody consisted of a dichotic sequence of four tones - each tone lasting 100 ms. The sequence of pitches differed between ears to reach a high task difficulty as in the other conditions. We chose this complex presentation to keep a clear-cut differentiation to the pitch discrimination task. In about half of the trials, the sequence of pitches was strictly descending on both sides. The tonal range was between 3068 and 304 Hz, the descent of pitches was up to 20 semitones between tones and up to 23 semitones within a melody. In about the other half of the trials a pitch ascent up to three semitones occurred at one of both ears; the side where a frequency ascended alternated to avoid a lateralization effect. Unpublished pilot studies suggested that a pitch ascent was detectable only when the other pitches at the same ear descended notably and, at the other ear, pitch remained constant during the ascent. Pitch could ascent either after the first or after the second tone to maintain attention [29]. The test applied four different basic melodies in which a pitch could ascend or not ascend in order to match the number of four alternative stimuli in the pitch identification task. Because the study does not focus on pitch contour perception, we present here only two examples of the 16 possible melodies (four melodies × 2 target/no target × 2 left/right variations). For instance, in the left ear, pitches were 683, 724, 645, 215 Hz, and in the right ear pitches were 1149, 1149, 966, 304 Hz listed in temporal succession. In this example, the participant should detect the pitch ascent from 683 to 724 Hz. A melody without pitch ascent could be left 683, 645, 608, 215 Hz, right 1149-1024-966-304 Hz. The investigator instructed the participants to press their index finger after a pitch ascent; they did not indicate whether after the first or second pitch or whether at the left or right ear a pitch ascended.

In the localization task, the investigator instructed the participants to indicate the presentation side of the higher tones (918 Hz) as compared to the lower tones (900 Hz). The participants attended to two successive tones lasting 100 ms each; during the tone at one ear, the other side was silence. We selected this sequential binaural presentation because during dichotic tones most participants had serious difficulties to assign a pitch ascent to the correct ear (unpublished pilot study). During the localization task, a pitch ascent at the left or at the right ear during the first or second tone of two successive tones resulted in four combinations in which the participants had to respond. In few trials, both tones were high or low. The participants pressed the farthest left or the farthest right key or both keys of the 4-keypad to indicate the side where the higher tone occurred.

For testing pitch discrimination, participants compared two successive tones in each trial. Each tone lasted 100 ms. The frequency of the first tone was always 1000 Hz; the frequency of the second tone either was constant 1000 Hz or increased to 1006, 1008, 1010, or 1012 Hz. Participants were instructed to press their index finger when the frequency ascended.

Comparisons of each test to an auditory baseline should level out activation from task-unspecific auditory perception. The baseline task asked the participants to press the index finger whenever a tone occurred. The frequency of the tone was always 1400 Hz; the tone lasted 200 ms.

### Experimental procedure

Prior to the measures, the investigator explained the tasks and fMRI procedures. The participants practiced each task one at a time until performance did not improve further in each task.

For scanning, foam material upholstery between the head and the head coil reduced head motion. The ambient light was dimmed. An icon indicated the task, the set of possible stimuli, and the expected responses using graphical elements. The icon was projected on a translucent screen at the participants' feet; the participants observed the projection through a mirror attached to the head coil. The icon remained visible during the entire task block to reduce visual switch and memory load effects and changed whenever the task changed. The soundcard of a computer produced sine wave tones that began and ended with 10 ms onset/offset ramps. The participants heard the tones via headphones, which contained no magnetic material but relied on the static MR field [30]. The headphones attenuated the scanner noise. A volume of 75 dB SPL allowed for comfortable listening. Optical fibers transferred responses on the keypad to a computer. STIMCO software from the MEG-Center, Tübingen, Germany, presented the auditory stimuli and icons and recorded the responses. A TTL trigger pulse synchronized stimulus presentation with fMRI scanning.

Neuroimaging contained four functional imaging sessions each lasting 10 min. The four tasks (pitch identification, pitch contour perception, tone localization, pitch discrimination) and baseline testing were presented in a block design. In one block, participants performed five trials of the same task or baseline testing; after five trials, the task changed. In each session, each of the four tasks and baseline testing recurred in four blocks. Thus, all sessions together comprised 80 trials of each task and of baseline testing. To level out long-term shifts, all sessions started and ended with a block of the baseline task. The order of the other blocks was pseudo-randomized within the sessions. The trial types within a block were pseudo-random such that the number of expected responses slightly differed between tasks and between participants within a variance of 10%.

In each trial, imaging was performed every 6 s with a silent break of 3.2 s. Participants heard the sound patterns between 1.5 and 1.8 s after scanning noise offset during the silent period; the stimulation onset was jittered by 300 ms to maintain the participants' attention. The stimulation during the silent period reduced interference from the scanner noise [31]. This "sparse sampling" technique increased signals within the Heschl's gyrus [32,33] and within the superior temporal plane [34]. During BOLD response assessment, blocks of five trials of the same task, each trial 6 s, covered 30 s in which the participants maintained in the same task-dependent mental set.

### fMRI scanning

Magnetic resonance imaging was performed on a 1.5 T scanner (Siemens Magnetom, Erlangen, Germany). For the sensitive detection of blood oxygenation level-dependent (BOLD) effects, single-shot triple-echo EPI was applied across the whole brain (TEs = 17, 43, and 68 ms, TA = 2.8 s, TR = 6 s, 90° flip angle, matrix size = 64 × 56, 30 slices per acquisition cycle, voxel size = 3.6 × 3.6 × 4 mm with 1 mm gap). Single-shot triple-echo EPIs enhanced the BOLD contrast by reducing distortions and dephasing in fMRI measurement [35]. For maximum volume coverage including prefrontal cortex and the entire cerebellum, the operator tilted slices occipito-caudally. The mean angle of tilt was about -20° from the axial position with an individually optimizing of coverage. The scanner noise amounted to 98 dB SPL without earmuffs (about 28 dB SPL dampening).

### Data analysis

Analysis of behavioral responses in the auditory tasks evaluated a possible effect of different test difficulty. False alarms were subtracted from hits to correct for random responses.

A procedure for multi-echo image data described from Mathiak et al. (2002) [36] was used to pre-process the functional scans. In short, by averaging across the time series, average image intensities S_TE _were determined at each voxel and echo time. The three echoes of each volume acquisition were averaged with the weights S_TE _x TE to maximize BOLD contrast-to-noise ratio in the combined image. All participants moved their head less than 3 mm during the entire measurement. Motion correction relied on parameter estimates as recommended by Speck and Hennig (2001) [37] and was obtained for the first echoes of each volume acquisition. The analysis based on the statistical standard procedure of SPM2 http://www.fil.ion.ucl.ac.uk/spm/.

Montreal Neurological Institute (MNI) space served as the anatomical reference for normalization (152 subjects template) [38]. To compensate for inter-subject variance in a small sample, Mikl et al. (2008) [39] recommended an extensive kernel for smoothing. Therefore, the Gaussian kernel reached 12 mm full-width at half-maximum. Statistical parametric mapping in SPM2 applied the general linear model on a block design. The investigators discarded the first volume of each session from analysis to account for saturation effects. To exclude effects of task switching, each first trial of blocks was discarded as well. The four conditions: pitch identification, pitch contour perception, tone localization, and pitch discrimination were modeled as boxcar functions folded with a canonical hemodynamic response function (bigamma function with 6 s peak-delay).

Analysis contrasted the BOLD activation of each test to baseline and compared the BOLD response during a task to each other task in a random effect analysis. Identical numbers of trials in each task facilitated the comparison of scans between tasks. The statistical maps of the second-level statistics were thresholded p < .05 corrected for family-wise error according to Gaussian random field theory [40]. For exploratory purposes and to reduce false negative findings, we additionally analyzed weaker effects compared to baseline applying lower thresholds (false-discovery rate FDR [41] and p <. 001 uncorrected threshold). A conjunction analysis of minimum T-values compared the activation between pitch identification vs. all other tests. A ROI analysis of the hypothesized IPS activation estimated differences between the hemispheres using a paired t-test on the β parameter.

The automated anatomical labeling (aal) toolbox of SPM2 [42] allowed identifying the activated regions. The relationship between the scores in pitch identification and the β means compared to baseline in the IPS was analyzed using Pearson's coefficient.

## Results

### Behavioral performance

Pitch identification (38%), pitch contour perception (29%), and localization (29%) were moderately and similarly difficult (F = 1.27, d.f. = 2, G-G = 0.7, p = .289) as intended in the construction of the tests. The performance in the discrimination task, however, was lower than in a previous behavioral experiment [2]. Probability of targets was 25% in pitch identification and 50% in the other tasks. Although the percentage of discrimination scores was 16% above chance of 50%, significant differences to pitch identification (t(15) = 17.7, p < .001) and tone localization (t(15) = 5.3, p < .001) emerged. As expected, the baseline condition was easy (90% correct responses). The number of correct responses in the pitch identification task correlated with activation in the left (r = .53, p = .032) but not in the right (r = .38, p = .142) IPS.

### fMRI data

**Figure 1:**
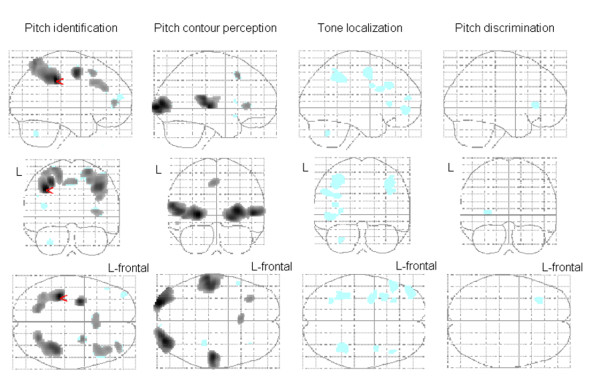
**Signal changes compared to baseline on the normalized SPM-glass brain**. The red arrow points to the only peak (x = -36, y = -34, z = 40) when the threshold p < .05 is FWE-corrected (see Table 1). *Pitch identification *activated the left intraparietal sulcus. Black regions show activation when the threshold p < .05 is FDR-corrected. In the azure regions emerged activation when the threshold is uncorrected p < .001. Table 1 displays a detailed description of regions activated after FWE- and FDR-correction. L = left hemisphere.

**Figure 2:**
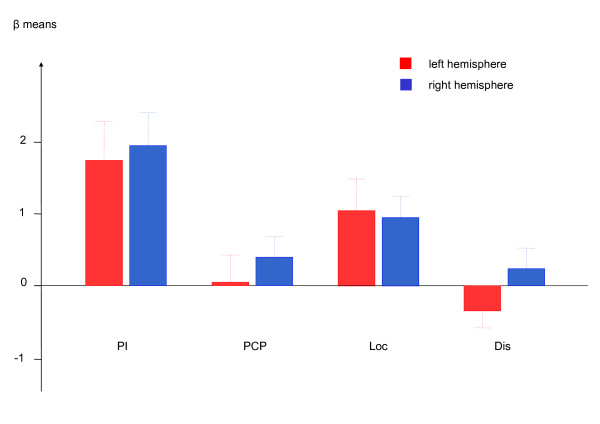
**Contrast estimations (± SE) in the left and right intraparietal sulcus during four auditory tasks**. *Pitch identification *clearly led to the strongest response. *Tone localization *activated the intraparietal sulcus as well. Lateralization effects failed significance. *Pitch identification *= PI; *pitch contour perception *= PCP; *tone localization *= Loc; *pitch discrimination *= Dis.

As compared to the auditory baseline task (key-press to each tone), pitch identification activated the left IPS at the FWE corrected threshold. At the FDR corrected threshold activation in the premotor regions, the right DLPFC, medial frontal gyrus, and medial frontal lobe reached significance. During pitch contour perception, the STG, the visual cortex, the left medial frontal gyrus and the insula showed an increased BOLD effect as compared to baseline applying FDR correction. In the others tasks, effects emerged only during a less restrictive threshold (p < .001 uncorrected). Tone localization yielded activation as compared to the baseline tonal task in the IPS among other regions. During both tone localization and pitch discrimination, low activation occurred in the medial frontal gyrus. Figure 1 and Table 1 display the activation patterns in detail. A ROI analysis on the hypothesized activation in the IPS revealed no lateralization effect between left and right hemispheres (t = 0.8, df = 15, p = .413). Figure 2 displays the activation in the IPS during each auditory task.

**Table 1:** Hemodynamic activation during *pitch identification *and *pitch contour perception *compared to baseline testing.

Anatomical substrate	T-score	MNI coordinates			Cluster size	Brodmann area
	**maximum**	**x**	**y**	**z**	**(ml)**	

*Pitch identification*						

L intraparietal sulcus	7.7*	-36	-34	40	8.8	40

R intraparietal sulcus	6.2	40	-40	44	10.8	40

L premotor area	6.7	-30	-6	50	2.2	6

R premotor area	5.0	24	-2	52	0.9	6

R dorsolateral prefrontal cortex	4.7	40	20	36	3.1	9

R medial frontal gyrus	4.7	4	18	50	1.3	8

R medial frontal lobe	4.6	34	48	8	0.8	10

						

*Pitch contour perception*						

L secondary visual cortex	6.7	-26	-96	2	6.9	18

R secondary visual cortex	6.4	30	-88	6	9.6	18

L superior temporal gyrus	5.8	-62	-30	14	7.1	41

R superior temporal gyrus	6.2	52	-22	4	3.5	41

L medial frontal gyrus	4.6	-4	18	48	0.6	8

L insula	4.5	-32	32	4	0.7	47

*p < .05 FWE corrected. All values p < .05 FDR corrected, T > 4.4. L/R = left/right.

**Figure 3:**
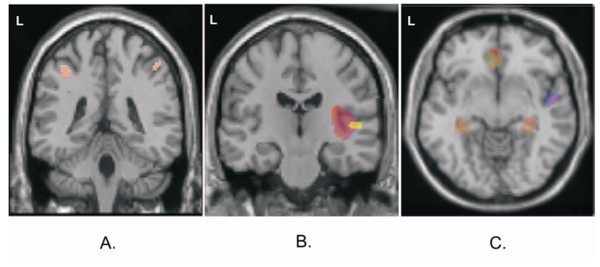
**Comparisons between the auditory tests using MNI coordinates of local maximum hemodynamic effects (T > 7.0, p < .05 FWE-corrected)**. The model subtracted activity during the baseline task before comparison. **A**. *Pitch identification *activated the intraparietal sulci (*red*; left x = -36, y = -38, z = 46; right x = 48, y = -42, z = 52) in comparison to *pitch discrimination*. **B**. *Pitch contour perception *led to higher activity in the right superior temporal gyrus (x = 50, y = -20, z = 6) as compared to all other auditory tests (*violet*); the cluster was more extended to lateral aspects as compared to *pitch identification *(*red*) and *discrimination *(*yellow*). **C**. Transversal view on z = -8. *Pitch contour perception *(*red*) and *discrimination *(*green*; *yellow *= both tests) activated the hippocampus (x = ± 32, y = -32) and the rostral anterior cingulate cortex (x = 2, y = 34) stronger than *pitch identification*. *Pitch contour perception *activated the right superior temporal gyrus (x = 36, y = 8) stronger than *pitch identification *and *tone localization *(*violet *= both tests; *blue *= localization).

Bilateral clusters emerged in ventral parts of the IPS during pitch identification in comparison to pitch discrimination (Figure 3a). The conjunction analysis revealed no significant difference between pitch identification and all other tests at the FWE-corrected threshold. However, peak T values of 6.1 at the left IPS (p = 8.8 × 10^-6 ^uncorrected threshold; × = -42, y = -30, z = 46; cluster size 5.2 ml) and 5.1 at the left IPS (p = 6.2 × 10^-5 ^uncorrected threshold; × = 44, y = -42, z = 48; cluster size 5.0 ml) indicated that pitch identification is the best candidate for activating the IPS.

Concerning activation during the other tasks, pitch contour perception increased the BOLD response in the caudal-ventral part of the right STG compared to the three other tasks (Figure 3b and 3c). Pitch contour perception and pitch discrimination activated the rostral anterior cingulate cortex (rACC) and the hippocampus more than pitch identification did (Figure 3c).

## Discussion

The findings support the hypothesis that auditory numerosity processing is associated with the IPS. Activation in the IPS during pitch identification may be the auditory counterpart of numerosity processing in the visual domain [5,11-13]. The resemblance of findings in the auditory and visual domain as well as a lack of activation in sensory auditory regions in comparison to the baseline suggests that IPS activity reflects the processing of supra-modal features. Left-sided lateralization during numerosity processing as in some visual studies was not replicable [12,16]. Thus, the neural differentiation between numerosity and magnitude processing remains unclear. However, the accuracy of pitch identification may induce a lateralization effect: only activation in the left IPS correlated with behavioral responses.

Further effects in comparison to the baseline were low. Labeling during pitch identification may induce activation in the DLPFC [3,43,44]. Arousal in the IPS but not in the DLPFC does not support the hypothesis that the IPS acts as a part of a working-memory related fronto-parietal network [45,46]. A response set with four instead of one alternative might contribute to the additional involvement of pre-motor area and the medial frontal lobe [47,48]. A tendency to activation in the medial frontal gyrus seems to characterize all auditory tasks though the activated hemispheres differ. The medial frontal gyrus might respond to the high difficulty of the tasks compared to simply hearing a tone in the baseline because this region is involved in error monitoring [49]. The wider variety and duration of tones in the pitch contour perception task were associated with higher activity in the auditory cortex. Processing similar to the perception of the simple tone in the baseline condition may have offset effects in discrimination and localization. The present data could not reveal a systematic bias due to dichotic stimulation. The activation patterns of the both tasks applying dichotic stimulation - pitch contour perception and localization - differed (STG vs. tendencies to IPS activation).

Pitch identification tended to differ from all other auditory tasks regarding IPS activation. IPS activity during localization reduced the contrast effect. The simultaneous variations of pitches and loci represented many alternatives and, thus, the localization task may elicit numerosity processing as well. Numerosity processing may add up stimuli independent from heterogeneous classifications [50]. Differential cerebral activation in the IPS validates the distinction between pitch identification and pitch discrimination in a behavioral study using the same tasks [2].

The study revealed two neural networks independent from pitch identification. One network was located at the right STG, which was activated during pitch contour perception as compared to all other tasks. A higher number of applied tones in each trial [18] or a higher range of pitches [51] may have stimulated the STG. A lack of effect in the STG but activation in the IPS during the pitch identification task suggest that pitch identification and its neural base is not associated to the diversity of stimulation.

Activation in the hippocampus and rACC during pitch contour perception and pitch discrimination compared to pitch identification suggested a further neural network. Instructions in these tasks emphasized that a false alarm reaction would lower the performance score. In animal experiments, a hippocampus-rACC network was associated with learning to avoid aversive stimuli [52,53]. The rACC may be involved in monitoring and coping with errors [54,55] in humans. However, possible effects of error monitoring were an accidental finding and not subject of the present study.

## Conclusions

Pitch identification is distinguishable from pitch discrimination on the base of IPS activity. In contrast to pitch identification, activation in an ACC-hippocampus network characterized pitch discrimination while a higher diversity of tonal stimulation increased the activation in sensory auditory regions. The processing of numerous pitches activates the IPS as numerosity processing of visual stimuli in previous studies did. Thus, IPS activity during pitch identification may be the auditory counterpart of numerosity processing in the visual domain.

## Competing interests

The authors declare that they have no competing interests.

## Authors' contributions

Both authors (MS and KM) made equally contributions to conception and design, analysis and interpretation of data, and drafting the manuscript. Both authors read and approved the final manuscript.

## Author's information

The first author wrote his doctoral thesis about effects of psychotropic drugs. He thought that the assessment of basic auditory effects in psychopharmacologic research was incomplete. Thus, he developed an auditory test battery. Prof. KM who had conducted several fMRI projects invited MS to participate in his research group in order to combine perception testing, psychopharmacology, and fMRI technique.
